# Compression of the Left Atrium and Pulmonary Veins Due to Ascending and Descending Aortic Aneurysms

**DOI:** 10.21470/1678-9741-2018-0395

**Published:** 2020

**Authors:** Muzaffer Kahyaoglu, Cetin Gecmen, Mehmet Altug Tuncer

**Affiliations:** 1 Department of Cardiology, Umraniye Education and Research Hospital, Istanbul, Turkey.; 2 Department of Cardiology, Kartal Kosuyolu Heart & Research Hospital, Istanbul, Turkey.; 3 Department of Cardiovascular Surgery, Kartal Kosuyolu Heart & Research Hospital, Istanbul, Turkey.

**Keywords:** Aged, 80 and over, Pulmonary Veins, Aorta, Thoracic, Mediastinum, X-Rays, Aortic Aneurysm, Echocardiography, Heart Atria, B

## Abstract

An 89-year-old female patient presented to our cardiology outpatient clinic complaining of shortness of breath and back pain. Chest X-ray demonstrated a widened mediastinum. Transthoracic echocardiography showed an ascending aortic aneurysm and the modified apical 5-chamber view showed that left atrium was compressed between the ascending and descending aortas. Color Doppler turbulence was also seen in the compressed area. A contrast-enhanced chest computed tomography scan in axial and coronal planes showed that left atrium and pulmonary veins were compressed by ascending and descending aortic aneurysms. Herein, we illustrated this rare condition diagnosed by transthoracic echocardiography in combination with computed tomography.

**Table:** 

Abbreviations, acronyms & symbols	
TAA	= Thoracic aortic aneurysm

## INTRODUCTION

Thoracic aortic aneurysm (TAA) is usually asymptomatic and incidentally detected by various diagnostic modalities. However, TAA may present with dissection or rupture, or chronically, with symptoms due to compression of the surrounding structures. Herein, we report a case of left atrium and pulmonary vein compression by ascending and descending aortic aneurysms.

## CASE REPORT

**Fig. 1:**
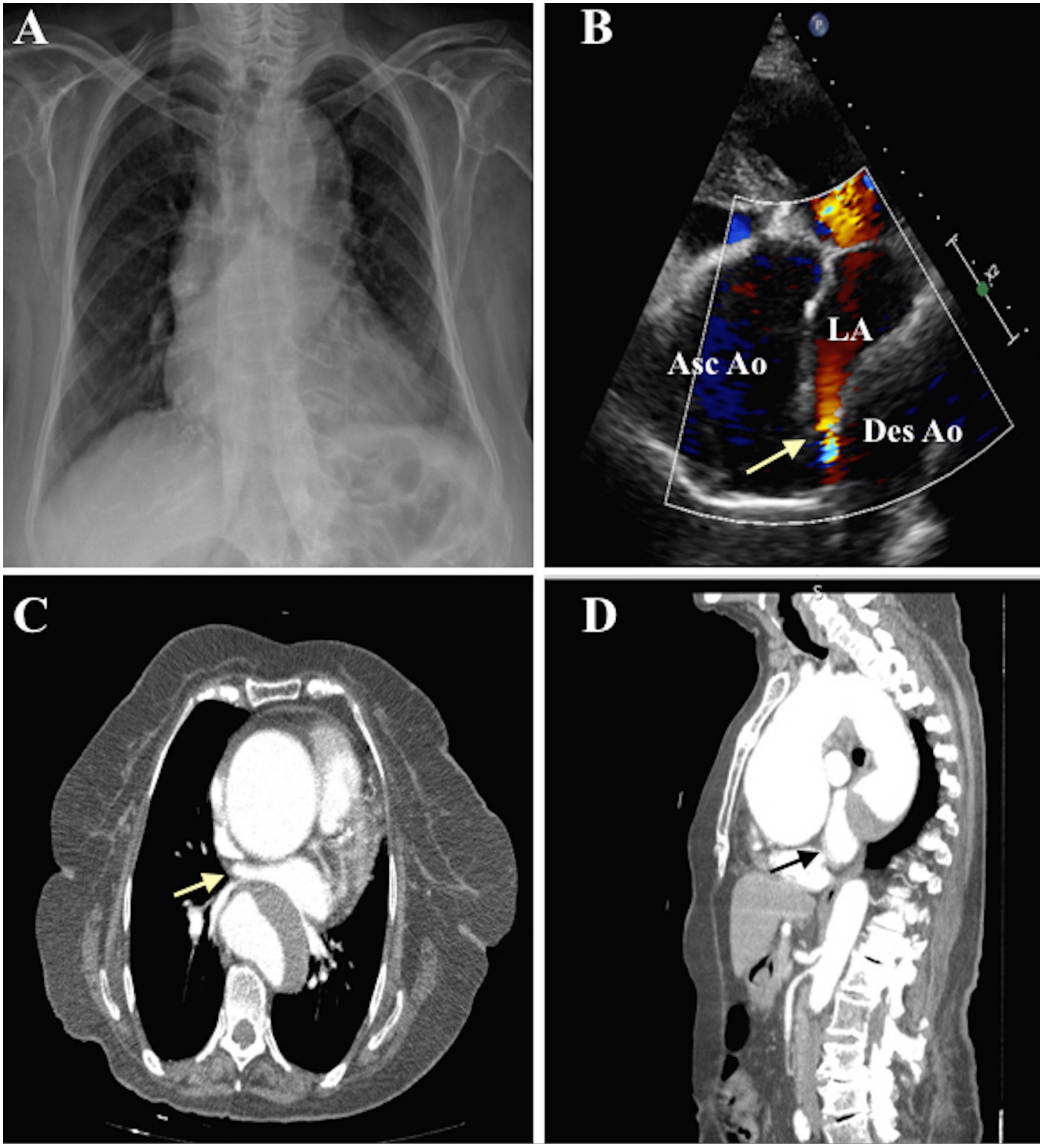
A) Chest X-ray demonstrated a widened mediastinum. B) Transthoracic echocardiography in modified apical 5-chamber view showed that left atrium was compressed between ascending and descending aortas, and color Doppler turbulence was seen in the compressed area (arrow). C) A contrast-enhanced chest computed tomography scan in axial plane showed that left atrium and pulmonary veins were compressed between ascending and descending aortic aneurysms. D) A contrast-enhanced chest computed tomography scan in coronal plane showed that left atrium and pulmonary veins were compressed between ascending and descending aortic aneurysms.

An 89-year-old female patient presented to our cardiology outpatient clinic complaining of shortness of breath and back pain. Her symptoms worsened over the last 6 months and the degree of dyspnea on admission corresponded to New York Heart Association class II-III. She also suffered from type 2 diabetes and non-regular hypertension. Upon physical examination, her blood pressure was 155/95 mmHg, heart rate was 78 bpm, and oxygen saturation was 91%. She also had an early diastolic murmur at the right sternal border. Laboratory findings were within normal limits. The electrocardiogram showed nonspecific T-wave changes. Chest X-ray demonstrated a widened mediastinum (Figure 1A). Transthoracic echocardiography showed an ascending aortic aneurysm and the modified apical 5-chamber view showed that the left atrium was compressed between the ascending and descending aortas. Color Doppler turbulence was also seen in the compressed area (Figure 1B). Left ventricular systolic function was normal, with ejection fraction of 65% and mild aortic regurgitation. A contrast-enhanced chest computed tomography scan in axial and coronal planes showed that left atrium and pulmonary veins were compressed by ascending and descending aortic aneurysms (Figures 1C and 1D). Surgical management was advised, but her family preferred a conservative treatment.

## DISCUSSION

TAA have an estimated incidence of at least 5 to 10 per 100,000 person-years^[1]^. The cause, natural history, and treatment vary depending on the location of the TAA. Aortic root or ascending aortic aneurysms are the most common (≈60%), followed by descending aorta aneurysms (≈35%) and aortic arch aneurysms (<10%)^[1]^. Most patients with TAA are asymptomatic, and the aneurysm is usually discovered incidentally on chest radiography, echocardiography, computed tomography, or magnetic resonance imaging^[2,3]^. Findings on physical examination such as aortic regurgitation may lead to new images and diagnosis of TAA. Aneurysms that produce symptoms are typically very large and are at an increased risk for rupture, which is associated with high mortality rates. When symptoms occur, patients may have chest or upper back pain, or symptoms related to compression of surrounding structures such as superior vena cava, trachea, bronchus, esophagus, left and right atrium or pulmonary veins. Occasionally, TAAs are associated with systemic embolization as a result of mural thrombus^[1,2,4]^.

Aortic root dilatation may lead to symptoms of dyspnea due to aortic regurgitation in ascending aortic aneurysm, and left atrium or pulmonary veins compression may cause dyspnea^[4,5]^. Compression of these structure may impair left vetricular diastolic filling, presented as shortness of breath. Occasionally, hemodynamic instability may develop as a result of decreased left ventricular filling^[4,6]^.

Compression of the left atrium or pulmonary veins due to type A aortic dissection, descending aortic aneurysm or pseudoaneurysm has been previously described. However, left atrial and pulmonary veins compressed by both ascending and descending aortic aneuryms is a rare condition.

In conclusion, we illustrated this rare condition diagnosed by transthoracic echocardiography in combination with computed tomography.

**Table:** 

Authors' roles & responsibilities	
MK	Substantial contributions to the conception or design of the work; or the acquisition, analysis, or interpretation of data for the work; drafting the work or revising it critically for important intellectual content; final approval of the version to be published
CG	Agreement to be accountable for all aspects of the work in ensuring that questions related to the accuracy or integrity of any part of the work are appropriately investigated and resolved; final approval of the version to be published
MAT	Final approval of the version to be published.
